# Machine-learning-based integration of temporal and spectral prompt gamma-ray information for proton range verification

**DOI:** 10.1016/j.phro.2025.100788

**Published:** 2025-06-02

**Authors:** Aaron Kieslich, Sonja M. Schellhammer, Alex Zwanenburg, Toni Kögler, Steffen Löck

**Affiliations:** a OncoRay – National Center for Radiation Research in Oncology, Faculty of Medicine and University Hospital Carl Gustav Carus, TUD Dresden University of Technology, Helmholtz-Zentrum Dresden-Rossendorf, Dresden, Germany; b Helmholtz-Zentrum Dresden-Rossendorf, Institute of Radiooncology – OncoRay, Dresden, Germany; c Zittau/Görlitz University of Applied Sciences, Faculty of Natural and Environmental Sciences, Zittau, Germany; d German Cancer Consortium (DKTK) Partner Site Dresden, and German Cancer Research Center (DKFZ), Heidelberg, Germany; e National Center for Tumor Diseases (NCT), NCT/UCC Dresden, a partnership between DKFZ, Faculty of Medicine and University Hospital Carl Gustav Carus, TUD Dresden University of Technology and Helmholtz-Zentrum Dresden-Rossendorf (HZDR), Germany; f Department of Radiotherapy and Radiation Oncology, Faculty of Medicine and University Hospital Carl Gustav Carus, TUD Dresden University of Technology, Dresden, Germany

**Keywords:** Proton radiotherapy, Treatment verification, Prompt gamma-ray timing, Machine learning, Multivariate modelling

## Abstract

**Background and Purpose:**

Prompt gamma-ray timing (PGT) and prompt gamma-ray spectroscopy (PGS) are non-invasive techniques for dose delivery monitoring in proton radiotherapy. Integrating PGT and PGS into a unified data analysis framework may improve proton range verification by incorporating both temporal and spectral information from prompt gamma-ray events. This study evaluates the effectiveness of this integration for enhancing the accuracy of proton range verification using machine-learning.

**Material and Methods:**

A homogeneous phantom was irradiated with 162 and 225 MeV static and scanned proton beams. Air cavities of 5, 10 and 20 mm were introduced to simulate anatomical variations. The energy and time of arrival of prompt gamma rays were measured using a PGT detector. 2-dimensional time-energy spectra were extracted for 1,440 proton spots. Different feature sets (energy-only, time-only, energy-restricted time, image) were computed. These feature sets were used by four different machine-learning models to predict range shifts. Model performance was assessed using the root mean square error (RMSE).

**Results:**

Time-only and combined time-energy feature sets exhibited good performance with RMSE values of 3 to 4 mm, consistent with previously developed models. Energy-only and image features led to poorer performance with RMSE values exceeding 5 mm. The integration of energy-only features did not improve prediction accuracy compared to exclusively using time-only features.

**Conclusion:**

While spectral information did not contribute additional value for determining proton beam range shifts in the investigated setup, the findings show that temporal information alone is sufficient to perform accurate proton range verification.

## Highlights

• Spectral data from uncollimated prompt gammas for proton range is not yet explored.
• A homogeneous PMMA phantom with air cavities of different thicknesses was irradiated.
• Machine-learning models using temporal and spectral data were developed and compared.
• No added value for proton range shift prediction using spectral data was observed.
• Temporal data was sufficient for accurate proton range verification.

## 1 Introduction

Proton radiotherapy provides precise dose delivery due to the Bragg peak, allowing high tumour doses while sparing healthy tissue [1]. However, this precision makes it sensitive to anatomical changes, highlighting the need for proton range verification to ensure accurate treatment delivery [2].

Prompt gamma rays have been widely used for proton range verification through prompt gamma-ray imaging (PGI) [3], [4], [5], [6], [7], [8], [9], prompt gamma-ray spectroscopy (PGS) [10], [11], and prompt gamma-ray timing (PGT) [12], [13], [14], [15].

PGS measures the energy of gamma rays emitted at a specific depth within the patient. The photon yield correlates with the proton energy and thus with the remaining range of the particles at that location. However, PGS requires heavy collimators to measure the gamma rays emitted at a specific depth, which may hinder their broader clinical applicability.

PGT uses the temporal distribution of promptly emitted photons to assess the range of the protons. Since PGT does not require collimated detectors, the system is lighter, requires less space and can therefore be better integrated into existing treatment systems [16].

Since both prompt gamma-ray energy and detection time are range-dependent, integrating these domains might provide complementary information to improve proton range verification [16]. Therefore, this study investigates the integration of temporal data from PGT with additional spectral information. Specifically, we evaluate the performance of various feature classes, including time-only features, energy-only features, energy-restricted time features, image features, and a combined feature set, across both static and scanned irradiation data. By utilising all information measured by a PGT detector and employing various machine-learning models, the aim is to maximise the accuracy of proton range assessments to support the clinical application of prompt gamma-ray-based proton treatment verification using an uncollimated detector system.

## 2 Material and methods

### 2.1 Experimental setup

**Fig. 1:**
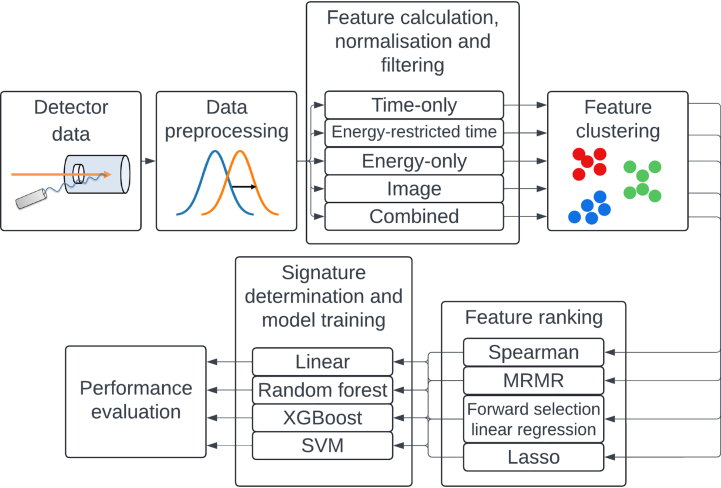
Overview of the workflow. The process begins with the collection and preprocessing of the detector data. This is followed by feature extraction, normalisation, and filtering. Feature clustering and ranking are conducted using various feature-selection algorithms. The final signature is determined, and the model is trained using various machine-learning algorithms. All steps excluding the performance evaluation were performed on the training set. The model performance was investigated on the test sets. Abbreviations: MRMR, minimum redundancy maximum relevance; SVM, support vector machine; XGBoost, eXtreme Gradient Boosting.

**Fig. 2:**
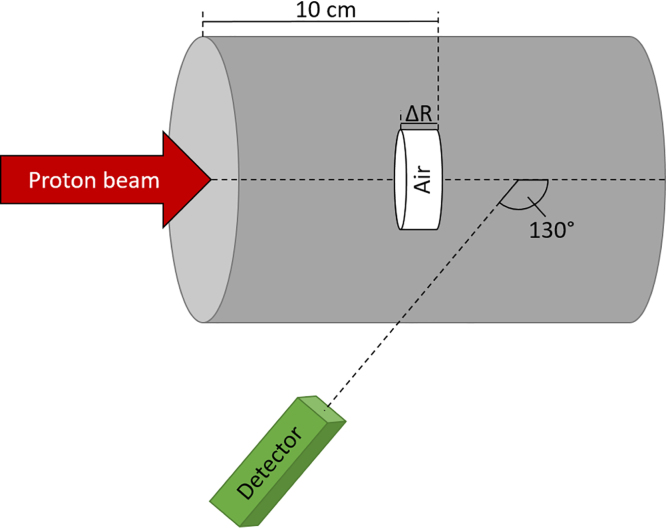
Schematic representation of the experimental setup. The setup consists of a cylindrical acrylic glass phantom (diameter: 15 cm, length: 40 cm) with adjustable air cavities (ΔR = 0, 5, 10, 20 mm), and a CeBr_3_-based detector for measuring the temporal and spectral characteristics of prompt gamma rays. The beam was delivered in static and scanned modes with energies of 162 MeV and 225 MeV, and prompt gamma rays were recorded at a backward angle of 130°. The detector was positioned such that its axis intersected the beam axis at half the depth of the proton range. Further details on the experimental setup and procedures can be found in [14].

The general study workflow is illustrated in Fig. 1, and was implemented using the Snakemake framework [17]. The experimental data used for this study were obtained from PGT measurements described in detail by Werner et al. [13], [14]. A schematic representation and a photo of the experimental setup can be found in Fig. 2 and Supplementary Figure S1, respectively. A homogeneous cylindrical phantom comprised of acrylic glass (${\left[{\text{C}}_{5}{\text{O}}_{2}{\text{H}}_{8}\right]}_{\text{n}}$) was irradiated with two proton energies: 162 and 225 MeV. To mimic anatomical variations leading to range deviations, air cavities of varying thickness ΔR (0, 5, 10, 20 mm) were incorporated into the phantom.

In the initial measurement series, a static pencil beam with $10^{9}$ protons per spot was used. 100 spots were irradiated per measurement. The first 30 spots were excluded from the analysis due to the phase oscillation effect [15]. In the subsequent series, a pencil beam scanning layer with 225 spots, arranged as a grid covering a quadratic 8 × 8 cm${}^{2}$ field, was irradiated 22 times, with each spot containing $10^{8}$ protons. The five central spots per layer were further analysed. Further details on the experimental setup and its rationale can be found in Supplementary material A.

The data were split into training and test sets, with the first 50 static spots per measurement used for training and the last 20 for testing. Scanned irradiations were exclusively used for testing to establish an independent test set and assess model generalisation across irradiation modes. To simulate the event count of a clinically envisioned eight-detector PGT system, scanned spots from eight consecutive layers were accumulated, achieving comparable counting statistics to static spots. The data were divided into three subsets: (i) 162 MeV, (ii) 225 MeV, and (iii) a combined energy-overarching dataset (162 & 225 MeV), with (i) and (ii) representing single-energy approaches and (iii) enabling predictions across both distinct energies.

### 2.2 Data preprocessing

**Fig. 3:**
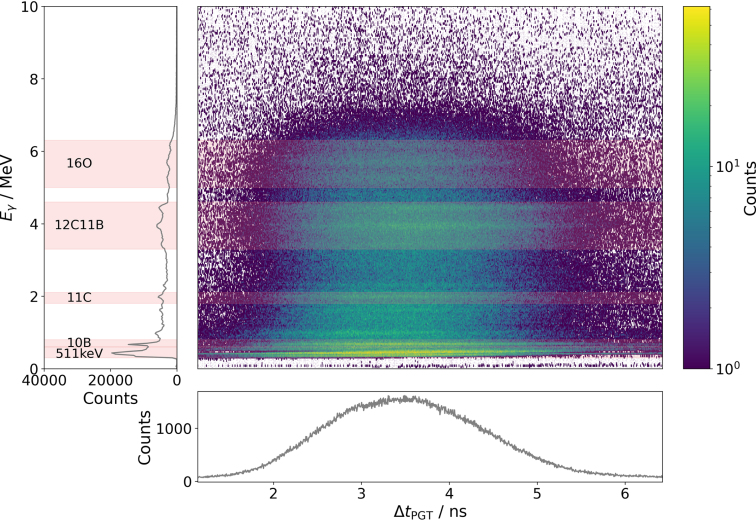
The processed 2D spectrum of the gamma-ray energy $E_{\gamma }$ as a function of the trigger time with respect to the cyclotron radio frequency $\Delta t_{\text{PGT}}$ (centre), energy spectrum (left), and time spectrum (bottom) of all static spots for the 162 MeV proton energy accumulated without introduced air cavity. The analysed gamma-ray energy ranges (see Table 1) are highlighted in red.

The preprocessing of the experimental data was conducted using the detector signal correction procedure outlined in [14]. Further data preprocessing steps are detailed in the Supplementary material B. In addition to the preprocessing, the generated 2-dimensional (2D) energy–time spectra were subdivided into several energy ranges (see Table 1). An example of a preprocessed spectrum is depicted in Fig. 3.

**Fig. 4:**
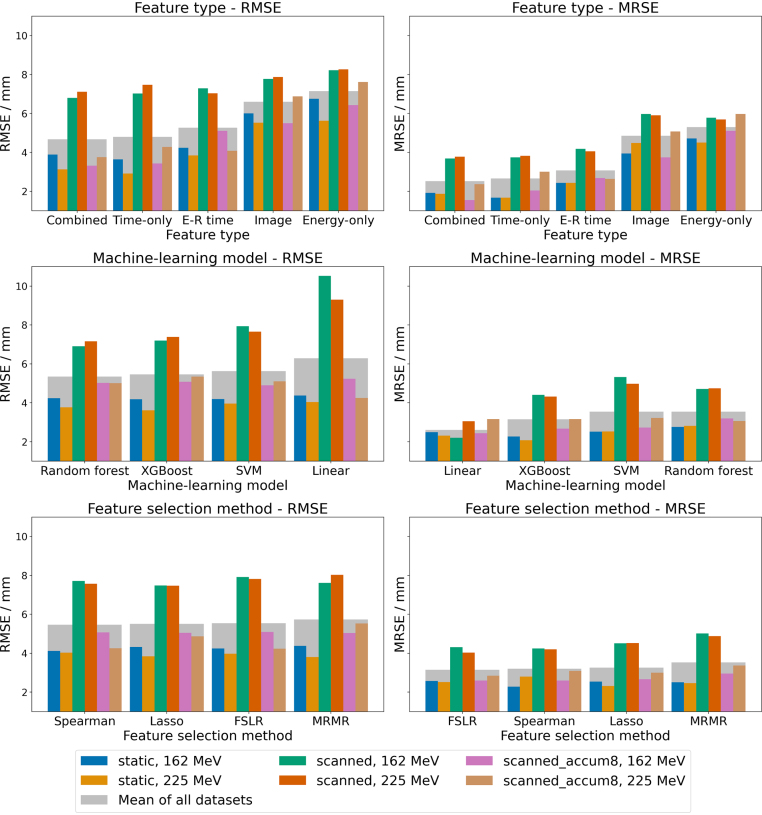
Aggregated performance of different modelling configurations for the energy-overarching experiment (iii) for the metrics RMSE and MRSE. The figure consists of subplots, each representing a different setup parameter: feature type (top), machine-learning model (mid) and feature-selection method (bottom). Each subplot compares the median performance of all models with the specified setup parameter for various test dataset combinations, with unique colours representing different datasets. The x-axis of each subplot shows the categories of the setup parameter sorted by the mean performance for all datasets (grey bars in the background), while the y-axis represents the RMSE (left) and MRSE (right) values. Abbreviations: E-R, energy-restricted, FSLR, forward selection linear regression; MRMR, minimum redundancy maximum relevance; MRSE, mean range shift error; RMSE, root mean squared error; SVM, support vector machine; XGBoost, eXtreme Gradient Boosting.

### 2.3 Feature extraction and processing

Five feature sets were defined to characterise the preprocessed spectra.

The time-only features capture key characteristics of the 1D time spectrum. These characteristics quantify, e.g. the distribution’s fall-off position, width, and intensity profile. For this feature set, we used the same features as described in [15].

The energy-restricted time feature set contains the time-only features calculated from the 1D time spectra restricted by the energy ranges specified in Table 1.

The energy-only features quantify the number of detected gamma-ray events for specific energy ranges (see Table 1), including the ratios of event numbers between these ranges. Additionally, these features were calculated across 5 time intervals, each spanning 230 time bins (=^ 1.06 ns), which effectively divides the spectrum into segments corresponding to different moments during proton irradiation. This segmentation acts as a form of virtual spatial collimation, conceptually similar to the collimation performed in PGS, allowing to capture both temporal and spectral dynamics of the prompt gamma-ray emissions. Details on the energy-only feature definitions can be found in the Supplementary material C.

**Table 1:** Overview of energy regions with associated nuclear interactions and energy ranges. The energy ranges include single and double escape peaks where applicable. The subdivision of the energy–time spectra into several energy ranges allowed for a focused examination of particular characteristics within the spectra.

Name	Nuclear interaction	Energy range / MeV
511 keV	Positron annihilation	0.3–0.6
10B	${}^{12}\text{C}{\left(p,2p\;n\right)}^{10}{\text{B}}^{*}$	0.6–0.8
11C	${}^{12}\text{C}{\left(p,p\;n\right)}^{11}{\text{C}}^{*}$	1.8–2.1
12C+11B	${}^{12}\text{C}{\left(p,p\right)}^{12}{\text{C}}^{*}$, ${}^{16}\text{O}{\left(p,p\;\alpha \right)}^{12}{\text{C}}^{*}$,	3.3–4.6
	${}^{12}\text{C}{\left(p,2p\right)}^{11}{\text{B}}^{*}$	
16O	${}^{16}\text{O}{\left(p,p\right)}^{16}{\text{O}}^{*}$	5.0–6.3

**Table 2:** RMSE values with 95% confidence intervals for various experiment configurations and test datasets. For the single-energy subsets (i) and (ii), data from the irradiations with proton energies of 162 MeV and 225 MeV were used for model development, respectively. The energy-overarching approach (iii) combines data from both 162 MeV and 225 MeV irradiations. The specific model configuration, including feature type, feature-selection method, and machine-learning model, was selected based on the best performance during cross-validation. The datasets are categorised based on the proton energy used for irradiation (162 MeV and 225 MeV) and the type of spots analysed (static vs. scanned). The specific datasets include static spots (static), single scanned spots (scanned), scanned spots accumulated over eight layers (scanned_accum8). Confidence intervals are indicated in brackets and calculated from 1000 bootstrap samples. Abbreviations: MRMR, minimum redundancy maximum relevance; RMSE, root mean squared error; XGBoost, eXtreme Gradient Boosting.

Subsets	(i) 162 MeV	(ii) 225 MeV	(iii) 162 & 225 MeV
Feature type	Combined	Combined	Time-only
Feature selection method	Lasso	MRMR	Lasso
Machine-learning model	XGBoost	XGBoost	XGBoost
Dataset	RMSE / mm		
(spot type, proton energy / MeV)			
static, 162	3.90	–	3.17
	(3.30–4.50)		(2.59–3.65)
static, 225	–	1.99	2.68
		(1.54–2.37)	(2.27–3.15)
scanned, 162	6.48	–	6.56
	(6.08–6.92)		(6.08–6.93)
scanned, 225	–	7.11	7.11
		(6.60–7.53)	(6.79–7.59)
scanned_accum8, 162	3.63	–	3.55
	(2.80–4.50)		(2.96–4.20)
scanned_accum8, 225	–	4.74	5.09
		(3.30–6.05)	(4.02–6.32)

The image features capture the texture, shape, and intensity patterns within a 2D image and are widely applied in the context of Radiomics [18]. These features were calculated for the full and energy-restricted (see Table 1) 2D energy–time spectra using the Medical Image Radiomics Processor (MIRP, version: 1.3.1) [19]. Details on the parameters used for the image feature extraction is listed in Supplementary Table S1.

The combined feature set contains the time-only, energy-only, energy-restricted time and image features, incorporating both temporal and spectral information from the prompt gamma-ray data.

To ensure that the features in the final models are comparable, robust across different irradiation settings and to reduce dimensionality, feature normalisation, filtering and clustering was performed. Detailed descriptions of these processes are provided in the Supplementary material D. The parameters used for feature clustering are listed in Supplementary Table S2.

### 2.4 Feature ranking, signature determination and final model development

We ranked the importance of cluster representative features using various feature-selection methods, including maximum relevance minimum redundancy (MRMR) [20], Spearman correlation, forward selection linear regression, and Lasso regression [21], each providing a distinct approach to feature importance assessment.

For model development, signature sizes from one to ten were incrementally tested, each composed of the top-ranked features. Performance was evaluated via 3-times repeated 3-fold cross-validation on the training set, using the RMSE metric across multiple machine-learning models: a multivariate linear model, a random forest [22], an eXtreme Gradient Boosting (XGBoost) tree [23], and a support vector machine (SVM) [24]. The signature achieving the lowest RMSE was selected and then used to train the final model on the entire training set. Given the five available feature sets, four feature-selection algorithms, and four model learners, a total of 80 modelling configurations were employed per subset (i), (ii) and (iii). The feature ranking, signature determination and final model development was supported by the “Fully Automated Machine Learning with Interpretable Analysis of Results” (FAMILIAR) framework [25]. The main parameters of FAMILIAR used for feature ranking, signature determination and final model training are listed in Supplementary Tables S3 and S4.

### 2.5 Performance evaluation

The performance of the final models was evaluated using the root mean squared error (RMSE). However, RMSE alone may not fully capture the model’s clinical relevance. Therefore, we introduced a new metric, the mean range shift error (MRSE), which provides a more clinically relevant assessment of the model’s accuracy in contexts where the aggregated predictions of multiple spots are used to detect treatment deviations [9], [26]. Let $y_{i}$ denote the true value of the ith air cavity thickness and ${\overset{ˆ}{y}}_{i,j}$ the jth predicted value for the same, with $M_{i}$ representing the number of predictions made for the ith air cavity and N the total number of air cavity thicknesses (here, N=4). The MRSE is defined as

(1) $$\text{MRSE}=\frac{1}{N}\overset{N}{\underset{i=1}{\sum }}\left|\frac{1}{M_{i}}\overset{M_{i}}{\underset{j=1}{\sum }}{\overset{ˆ}{y}}_{i,j}-y_{i}\right|,$$

which evaluates the mean absolute error between the true air cavity thickness and the average of the model’s predictions for each air cavity. In contrast, the RMSE is computed over all individual predictions as

(2) $$\text{RMSE}=\sqrt{\frac{1}{\overset{N}{\underset{i=1}{\sum }}M_{i}}\overset{N}{\underset{i=1}{\sum }}\overset{M_{i}}{\underset{j=1}{\sum }}{\left({\overset{ˆ}{y}}_{i,j}-y_{i}\right)}^{2}}.$$

## 3 Results

The integration of temporal and spectral prompt gamma-ray information for proton range verification has been evaluated using three subsets ((i)-(iii)) and various combinations of feature types, feature-selection methods and machine-learning models.

Our feature ranking process identified key predictors for proton range verification. For time-only features and using Lasso regression feature selection, robust mean absolute deviation (10-90%), entropy, and mean were most important (see Supplementary Figure S2), reflecting time spectrum width, complexity, and central tendency, all linked to proton time-of-flight. For energy-only features, the absolute intensity of the ${}^{16}\text{O}$ emission line, total spectrum intensity, and relative intensity of the 511 keV line were most predictive (see Supplementary Figure S3). Time-restricted peak ratios, assumed to act as virtual spatial collimation, were less relevant, likely due to lower statistical reliability. Absolute intensities performed better, as range shifts change the detector’s solid angle, affecting detected counts.

The RMSE values for the models with the lowest RMSE during cross-validation are summarised in Table 2. During cross-validation, the combined feature set was optimal for the single-energy approaches (i) and (ii), while the time-only feature set showed best results for the energy-overarching approach (iii). On the test sets, static spots consistently provided the highest accuracy across all datasets and approaches (RMSE between 1.99 and 3.90 mm). For single scanned spots, the RMSE values were comparable across the different subsets (RMSE between 6.48 and 7.11 mm). For scanned spots accumulated over eight layers (scanned_accum8), the RMSE values showed a notable improvement in all cases compared to the single scanned spots datasets. Comparable trends are present for the MRSE (Supplementary Table S5). The comparison of the RMSE and MRSE between the single-energy approaches and the energy-overarching approach showed no pronounced differences.

The average performance of different modelling configurations for the energy-overarching approach (iii) is illustrated in Fig. 4. Differences in average performance across all datasets between models based on time-only features and combined features were minimal, with RMSE and MRSE being 4.79 mm and 2.66 mm for time-only features compared to 4.67 mm and 2.53 mm for combined features. In contrast, models based on image and energy-only features consistently exhibited poorer performance, with RMSE values of 6.59 mm and 7.15 mm, and MRSE values of 4.85 mm and 5.29 mm, respectively.

Linear models exhibited, on average across all datasets, higher RMSE (6.29 mm) compared to other machine-learning models such as random forest, which achieved an average RMSE of 5.35 mm. In contrast, linear models outperformed the other machine-learning models in terms of MRSE, with an average value of 2.61 mm compared to 3.15 mm for XGBoost tree models. The performance gap between linear models and other machine-learning models was most pronounced for single scanned spots, where the RMSE reached 10.52 mm for linear models versus 6.90 mm for random forest models. Similarly, MRSE for single scanned spots was markedly lower for linear models at 2.21 mm compared to 4.41 mm for XGBoost tree models.

Differences between feature-selection methods were minor, with average RMSE values ranging from 5.46 mm (Spearman) to 5.73 mm (MRMR) and MRSE values ranging from 3.14 mm (forward selection linear regression) to 3.53 mm (MRMR), suggesting multiple similar solutions of finding accurate range predictors.

Detailed RMSE and MRSE values across all modelling configurations for the energy-overarching approach are presented in Supplementary Figures S4 and S5. The worst-performing model configurations for the energy-overarching approach based on the RMSE and the MRSE in each test dataset are depicted in Supplementary Figures S6 and S7.

## 4 Discussion

This study aimed to explore the integration of temporal and spectral prompt gamma-ray information for proton range verification. Our results revealed that, while the time-only features alone provided reliable predictions, the inclusion of spectral information did not result in noticeable improvements.

Image and energy-only features exhibited the worst performance. The limited value of spectral information compared to PGS systems [10], [11] likely stems from two main factors. First, mechanical collimation in PGS ensures that each detected gamma ray carries more range-relevant information by spatially filtering emissions from specific depths. In contrast, uncollimated PGT detectors capture gamma rays from the entire irradiation field, including the beam entrance and scattered events, diluting useful range information. Time windowing may not provide sufficient virtual spatial collimation, as the proton bunch time spread and detector resolution introduce uncertainty in correlating detection time with gamma-ray origin. Second, the generally low counting statistics typical of prompt gamma methods introduce high statistical uncertainties, further limiting the extraction of spectral features. This observation was further substantiated by applying the energy-only models to a test set of 10-fold accumulated static spots, which resulted in an RMSE of up to 2 mm (Supplementary Figure S8). This suggests that spectral information in PGT systems gains predictive value as counting statistics improve.

The impact of limited counting statistics became further evident when comparing static and scanned spots. Static spots outperformed scanned spots due to their higher intensity ($10^{8}$ vs. $10^{9}$ protons per spot). Aggregating scanned spots over eight layers reduced the performance gap, confirming counting statistics as the dominant factor. Beam shape, positional variations, and interaction effects are unlikely contributors, as geometry remained identical and only central spots were analysed. Lower counting statistics in scanned spots also increased uncertainty in preprocessing (e.g., background and phase-shift correction), affecting feature extraction and range shift predictions. These findings align with previous studies [14], [15], reinforcing counting statistics as a key limiting factor in PGT-based proton range verification.

Since limited counting statistics is the main factor of reduced spectral information and prediction accuracy in uncollimated systems, improving the detection efficiency and online processing capabilities of the PGT system is crucial. This can be achieved by integrating larger or more sensitive detectors with finer segmentation, fast individual readouts, and high-throughput data acquisition. This reduces dead time and pile-up while increasing the number of usable events. Additionally, the proportion of events with high range information should be increased through careful real-time event selection. Furthermore, aggregating nearby points within an energy layer of a treatment field can further improve counting statistics, with a slight loss of spatial resolution [9].

Although the combined feature set performed best in cross-validation for the single-energy approaches (i) and (ii), its advantage over the time-only feature set was minimal (Fig. 4, Supplementary Figures S4 and S5). Most selected features in the combined set were time-based (Supplementary Figure S9), and model predictions from both feature sets showed a strong Pearson correlation of 0.78 (Supplementary Figure S10), indicating that adding spectral information provided little additional predictive value beyond the temporal features alone.

Complex machine-learning models showed higher MRSE, suggesting that they overfit to the discrete and constrained range shifts introduced to the phantom. While tree-based models and SVMs achieve high precision (low RMSE) by restricting predictions close to the introduced range shifts, they produce asymmetric errors, lowering accuracy (high MRSE). In contrast, linear models provide more symmetrical prediction errors, resulting in lower MRSE. This makes them particularly relevant for model development on constrained training data. This effect was particularly pronounced for single scanned spots, where low counting statistics increased feature uncertainty, leading to greater prediction variability and higher RMSE in linear models. In contrast, complex machine-learning models, constrained by the training range, exhibited lower RMSE but asymmetric errors, increasing MRSE. However, as more diverse datasets from complex phantoms and Monte Carlo simulations become available, complex machine-learning models may improve by capturing nonlinear dependencies while avoiding overfitting on the constrained range shifts introduced to the phantom.

Previous studies have developed models to predict proton range shifts using uncollimated systems and temporal information [14], [15]. Schellhammer et al. [15] created multivariate linear models with time-only features, achieving an RMSE of about 4 mm. While analysing the same data, the present study improved prediction accuracy by 0.5 mm for static spots. Moreover, in contrast to Schellhammer et al. [15], the present work demonstrated that proton range can be effectively predicted by a single model across two distinct proton energies (162 MeV and 225 MeV). The proton energies provided comprehensive coverage of both typical clinical conditions (162 MeV) and methodological challenges (e.g. elevated neutron background) associated with higher energies (225 MeV), providing strong indication that the need for separate models tailored to specific proton energies might be eliminated in future clinical application. This improvement was achieved through a revised machine-learning pipeline, including iterative statistical background correction before phase-shift correction, normalising features based on a reference measurement (no air cavity) and applying feature clustering. Despite being incremental, the successful adjustments to the pipeline guide the development of future PGT models.

The main limitation of this study was the simplified experimental setup, restricted to two irradiation modes, two proton energies, central beam spots, and a homogeneous phantom. While this setup allowed for a controlled evaluation of spectral information, greater anatomical complexity could further complicate the relationship between range shifts and spectral information. Given the low performance of energy-only features, we do not expect spectral information to improve in complex phantoms or patient data without noticeable detector or experimental adjustments. Moreover, the optimal definition of energy features remains unclear. Studies have demonstrated the potential of deep learning in prompt gamma analysis [27], [28]. Deep-learning models might be able to automatically extract important features from both time and energy domains, potentially enhancing the proton range verification.

In conclusion, this study indicates that incorporating spectral information from prompt gamma-rays detected with an uncollimated detector system to the modelling of the proton range shift does not noticeably improve prediction accuracy compared to the temporal information alone. Using machine-learning on the temporal information is sufficient to make accurate predictions for proton range verification, offering a promising approach for future clinical applications.

## CRediT authorship contribution statement

**Aaron Kieslich:** Conceptualisation, Data curation, Methodology, Software, Validation, Formal analysis, Investigation, Writing – review & editing, , Writing original draft, Visualisation. **Sonja M. Schellhammer:** Conceptualisation, Resources, Writing – review & editing, Supervision. **Alex Zwanenburg:** Methodology, Writing – review & editing, Supervision. **Toni Kögler:** Conceptualisation, Project administration, Resources, Writing – review & editing, Supervision. **Steffen Löck:** Methodology, Writing – review & editing, Supervision.

## Code availability

The code used in this study is publicly available to facilitate reproducibility and further research. The code used to analyse the data is published on GitHub at https://github.com/oncoray/PGT_Modeling. This repository includes all scripts, functions, and data preprocessing steps used in this study.

## Declaration of Generative AI and AI-assisted technologies in the writing process

During the preparation of this work the authors used GPT-4o in order to improve the readability and language of the manuscript. After using this service, the authors reviewed and edited the content as needed and take full responsibility for the content of the publication.

## Declaration of competing interest

The authors declare that they have no known competing financial interests or personal relationships that could have appeared to influence the work reported in this paper.

## Appendix A Supplementary data

MMC S1 Supplementary material.

## References

[1] Jäkel O. (2009). Medical physics aspects of particle therapy. Radiat Prot Dosim.

[2] Lomax A. (2008). Intensity modulated proton therapy and its sensitivity to treatment uncertainties 2: the potential effects of inter-fraction and inter-field motions. Phys Med Biol.

[3] Smeets J., Roellinghoff F., Prieels D., Stichelbaut F., Benilov A., Fiorini C., Peloso R., Basilavecchia M., Frizzi T., Dehaes J. (2012). Prompt gamma imaging with a slit camera for real-time range control in proton therapy. Phys Med Biol.

[4] Peterson S., Robertson D., Polf J. (2010). Optimizing a three-stage compton camera for measuring prompt gamma rays emitted during proton radiotherapy. Phys Med Biol.

[5] Hyeong Kim C., Hyung Park J., Seo H., Rim Lee H. (2012). Gamma electron vertex imaging and application to beam range verification in proton therapy. Med Phys.

[6] Kasper J., Rusiecka K., Hetzel R., Kozani M.K., Lalik R., Magiera A., Stahl A., Wrońska A. (2020). The SiFi-CC project–feasibility study of a scintillation-fiber-based compton camera for proton therapy monitoring. Phys Med.

[7] Muñoz E., Barrientos L., Bernabéu J., Borja-Lloret M., Llosá G., Ros A., Roser J., Oliver J.F. (2020). A spectral reconstruction algorithm for two-plane compton cameras. Phys Med Biol.

[8] Richter C., Pausch G., Barczyk S., Priegnitz M., Keitz I., Thiele J., Smeets J., Stappen F.V., Bombelli L., Fiorini C., Hotoiu L., Perali I., Prieels D., Enghardt W., Baumann M. (2016). First clinical application of a prompt gamma based in vivo proton range verification system. Radiother Oncol.

[9] Berthold J., Pietsch J., Piplack N., Khamfongkhruea C., Thiele J., Hölscher T., Janssens G., Smeets J., Traneus E., Löck S. (2023). Detectability of anatomical changes with prompt-gamma imaging: First systematic evaluation of clinical application during prostate-cancer proton therapy. Int J Radiat Oncol Biol Phys.

[10] Verburg J.M., Seco J. (2014). Proton range verification through prompt gamma-ray spectroscopy. Phys Med Biol.

[11] Hueso-González F., Rabe M., Ruggieri T.A., Bortfeld T., Verburg J.M. (2018). A full-scale clinical prototype for proton range verification using prompt gamma-ray spectroscopy. Phys Med Biol.

[12] Golnik C., Hueso-González F., Müller A., Dendooven P., Enghardt W., Fiedler F., Kormoll T., Römer K., Petzoldt J., Wagner A., Pausch G. (2014). Range assessment in particle therapy based on prompt γ-ray timing measurements. Phys Med Biol.

[13] Werner T., Berthold J., Enghardt W., Hueso-González F., Kögler T., Petzoldt J., Richter C., Rinscheid A., Römer K., Ruhnau K. (2017). 2017 IEEE nuclear science symposium and medical imaging conference (NSS/MIC).

[14] Werner T., Berthold J., Hueso-González F., Kögler T., Petzoldt J., Römer K., Richter C., Rinscheid A., Straessner A., Enghardt W. (2019). Processing of prompt gamma-ray timing data for proton range measurements at a clinical beam delivery. Phys Med Biol.

[15] Schellhammer S.M., Wiedkamp J., Löck S., Kögler T. (2022). Multivariate statistical modelling to improve particle treatment verification: Implications for prompt gamma-ray timing. Front Phys.

[16] Pausch G., Berthold J., Enghardt W., Römer K., Straessner A., Wagner A., Werner T., Kögler T. (2020). Detection systems for range monitoring in proton therapy: Needs and challenges. Nucl Instrum Methods Phys Res A.

[17] Mölder F., Jablonski K.P., Letcher B., Hall M.B., Tomkins-Tinch C.H., Sochat V., Forster J., Lee S., Twardziok S.O., Kanitz A. (2021). Sustainable data analysis with snakemake. F1000Res.

[18] Van Timmeren J.E., Cester D., Tanadini-Lang S., Alkadhi H., Baessler B. (2020). Radiomics in medical imaging—“how-to” guide and critical reflection. Insights Imaging.

[19] Zwanenburg A., Löck S. (2024). MIRP: A python package for standardised radiomics. J Open Source Softw.

[20] Peng H., Long F., Ding C. (2005). Feature selection based on mutual information criteria of max-dependency, max-relevance, and min-redundancy. IEEE PAMI.

[21] Tibshirani R. (1996). Regression shrinkage and selection via the lasso. J R Stat Soc Ser B Stat Methodol.

[22] Ho T.K. (1995). Proceedings of 3rd international conference on document analysis and recognition.

[23] Chen T., Guestrin C. (2016). Proceedings of the 22nd ACM SIGKDD international conference on knowledge discovery and data mining.

[24] Cortes C., Vapnik V. (1995). Support-vector networks. Mach Learn.

[25] Zwanenburg A., Löck S. (2022).

[26] Pietsch J., Khamfongkhruea C., Berthold J., Janssens G., Stützer K., Löck S., Richter C. (2023). Automatic detection and classification of treatment deviations in proton therapy from realistically simulated prompt gamma imaging data. Med Phys.

[27] Munoz E., Ros A., Borja-Lloret M., Barrio J., Dendooven P., Oliver J.F., Ozoemelam I., Roser J., Llosá G. (2021). Proton range verification with MACACO II compton camera enhanced by a neural network for event selection. Sci Rep.

[28] Kozani M.K. (2024). Machine learning approach for proton range verification using real-time prompt gamma imaging with compton cameras: addressing the total deposited energy information gap. Phys Med Biol.

