# Supplementary material for: Machine-learning-based integration of temporal and spectral prompt gamma-ray information for proton range verification
Source: Phys Imaging Radiat Oncol. 2025 Jun 2;35:100788. doi: 10.1016/j.phro.2025.100788 (PMC12198029; doi:10.1016/j.phro.2025.100788)
Supplement: MMC S1 — Supplementary material. [file mmc1.pdf]

Supplementary material: Machine-learning-based integration of temporal and spectral prompt gamma-ray information for proton range verification

Supplementary material A: Experimental setup

The dataset used in this study originates from measurements previously described by Werner et al. [1] and Schellhammer et al. [2], ensuring that the data is well understood and thoroughly characterized.

A cylindrical acrylic glass phantom (diameter: 15 cm, length: 40 cm) was used for all measurements due to its well-characterized proton stopping power and its frequent use as a tissue-equivalent material in proton radiotherapy research. Acrylic glass provides a homogeneous medium, facilitating precise proton range measurements and comparison with theoretical models.

To investigate the impact of anatomical heterogeneities, air cavities with thicknesses of 0, 5, 10, and 20 mm were introduced within the phantom. These variations were selected to mimic clinically relevant air-filled spaces in the head-and-neck and lung regions, where range uncertainties are a known challenge in proton radiotherapy. Evaluating prompt gamma-ray detection under these conditions provides insights into the robustness of prompt gamma-ray timing (PGT) for range monitoring.

In the initial measurement session, a static pencil beam was aimed at the centre of the phantom. This setup involved pulsing the beam in defined spots, with each spot lasting 69 ms and separated by intervals of 72 ms, delivering 10^9 protons per spot. A total of 100 spots were included in each irradiation, although the data from the first 30 spots were not considered due to the phase oscillation effect [1].

The subsequent measurement session implemented pencil beam scanning, as detailed in reference [3]. In this session, each measurement involved the distal energy layer of a treatment plan that uniformly irradiated a cubic volume of 8 x 8 x 8 cm^3. This layer included 225 pencil beam spots corresponding to 225 scanning positions, with proton numbers per spot of approximately

1×10^8 in the centre mirroring typical clinical spot intensities. This energy layer was repeated 22 times per measurement. Since the range deviations are limited to the central area of the irradiation field due to the limited width of the air cavities, only the five central spots were selected for further analysis.

Both measurement sessions consisted of eight individual measurements covering the four different air cavity thicknesses and two beam energies (162 MeV and 225 MeV). The two proton energies were selected based on their relevance for both clinical and experimental purposes. The lower energy of 162 MeV closely resembles a clinically relevant treatment scenario, making it directly applicable to real-world therapy contexts. In contrast, the higher energy of 225 MeV was chosen to explore specific methodological challenges. At this higher energy, the proton bunch time spread is reduced, potentially improving the temporal resolution of PGT measurements. Additionally, 225 MeV allows for investigating whether PGT can effectively handle the increased neutron background that typically accompanies higher proton energies—an issue known to impact other prompt gamma-ray methods, as reported by Smeets et al. [4]. These two energies thus enable a comprehensive evaluation of the robustness and versatility of the PGT system.

Measurements were conducted using a detection unit that included a ø2”×2” $CeBr_3$ crystal by Scionix[1], a Hamamatsu[2] R13089-100 photomultiplier, and a U100 digital spectrometer by Target Systemelektronik[3]. The detector was positioned such that its axis intersected the beam axis at half the depth of the proton range.

The system's operational parameters included a detector trigger count rate of approximately 500 kcps, a dead time of 1 µs per event, a 5 % pile-up probability, and a proton beam current of 2 nA at the nozzle exit.

[1]Scionix Holland B.V., Regulierenring 5, 3981 LA Bunnik, Netherlands

[2]Hamamatsu Photonics K.K., 325–6, Sunayama-cho, Naka-ku, Hamamatsu City, Shizuoka Pref., 430–8587, Japan

[3]Target Systemelektronik GmbH & Co. KG, Heinz-Fangman-Straße 4, 42287 Wuppertal, Germany

Supplementary material B: Data preprocessing

The preprocessing of experimental data was conducted using the methodology outlined in [1]. Corrections were made for the drift in photomultiplier gain and the non-linearities in signal digitisation. The gamma-ray detection time relative to the accelerator radiofrequency and the gamma-ray energy were recorded to create a 2-dimensional (2D) spectrum for each proton spot.

The background region in our spectra was identified as the region with the lowest signal, which is assumed to represent mostly background noise. The summed 1D time spectrum of each irradiation was first smoothed using a median filter of size 7 and then a Gaussian filter of size 11. This helps to reduce noise and highlight broader trends in the data. The minimum value in the smoothed spectrum was taken as the baseline, representing the lowest level of signal, which is likely dominated by background noise. A threshold was then defined as a multiple of this baseline value with 1.05. The largest continuous region with counts below the threshold was then identified as the background region.

For each energy bin (e.g., for each bin along the energy axis) in the 2D spectrum, the mean value of the counts within the background region was calculated. This mean value represents the estimated background rate for that energy bin. Per energy bin, the estimated background rate for all spots of an irradiation was fitted using a linear function to account for increasing activation during irradiation. For each spot, the fitted background rate was subsequently used for further background correction.

The total number of background events expected in the entire spectrum was estimated by multiplying the fitted background rate by the total number of bins along the time axis (2048). This number was adjusted to be an integer using a probabilistic rounding method. The expected number of background events were randomly removed from the spectrum. This was done by generating random positions for the background events within the time axis and identifying the closest non-zero bins in the spectrum to these random positions. Then the counts get removed from these bins until the expected number of background events were removed.

This results in a background-corrected spectrum where the influence of the background noise was reduced. This method ensures that the background correction is both systematic and traceable, leveraging the statistical nature of the data.

As noted in earlier studies [3, 2], instabilities in the phase relationship

between proton bunch arrival and the accelerator's radio frequency need to be addressed. These instabilities include damped oscillations after changes in particle energy, oscillations in the mean time spectra of prompt gamma-rays, lasting about two seconds with amplitudes of a few hundred picoseconds. This is due to adjusting the acceleration voltage to minimise excess dose. Affected spots were excluded from the datasets, as mentioned in the main manuscript and performed in [3] and [2]. In addition, there is a drift in the proton bunch phase relative to the radio frequency, by several hundred picoseconds per hour, likely due to temperature-induced magnetisation changes in the cyclotron magnet. This can be corrected during preprocessing by aligning the timing distribution with a reference distribution.

The phase shift correction was conducted as previously described in [2]. However, it is important to note that in the current study, background correction was performed before the phase shift correction. Additionally, the rebinning of the shifted spectrum was performed using a probabilistic approach, ensuring the counts of the spectra remained in integer form. Maintaining the integer form of the counts ensures that the extracted features are well-defined (e.g. entropy). After background correction and phase drift correction, a global time window of 5.3 ns width (1152 time bins) starting at 1.14 ns (238th time bin) has been applied on the corrected spectrum to exclude the background region from the evaluation.

Supplementary material C: Energy-only features

The energy-only features aim to quantify the absolute and relative intensities of specific energy regions (ERs) within the 2D energy-time gamma-ray spectrum $h(t_i, e_i)$, as well as the peak intensity ratios between these ERs. The ERs considered include the full spectrum and specific ERs (511 keV, 10B, 11C, 12C11B, 16O). Three steps are performed to calculate the energy-only features:

- **Absolute and relative intensities**: For each ER, the total (absolute) intensity $I_{\text{abs,ER}}$ is calculated by summing the spectrum within the ER:

  $$I_{\text{abs,ER}} = \sum_{i \in \text{ER}} h(t_i, e_i)$$

  The relative intensity $I_{\text{rel,ER}}$ is then determined by normalising the absolute intensity to the total number of events in the full spectrum:

  $$I_{\text{rel,ER}} = \frac{I_{\text{abs,ER}}}{I_{\text{abs,full}}}$$

- **Peak ratios**: The ratio of absolute intensities between pairs of energy regions ER_1 and ER_2 is computed to capture the relative differences:

  $$R_{\text{ER}_1/\text{ER}_2} = \frac{I_{\text{abs,ER}_1}}{I_{\text{abs,ER}_2}}$$

- **Time slices**: The spectrum is divided into $n = 5$ equal-sized time intervals (slices). For each time slice j, the absolute intensities within each slice are calculated for each ER. Let $t_{i,j}$ denote the time bins within the j-th slice. The absolute intensity for ER within slice j is:

  $$I_{\text{abs,ER},j} = \sum_{i \in \text{ER}, i \in \text{slice } j} h(t_{i,j}, e_i)$$

  The peak ratios between pairs of ERs for each time slice j are then determined:

  $$R_{\text{ER}_1/\text{ER}_2,j} = \frac{I_{\text{abs,ER},j}}{I_{\text{abs,ER},j}}$$

  As a specific time interval is conceptually equivalent to a spatial collimation of the gamma-rays, these features represent the information used by PGS.

Supplementary material D: Feature normalisation, filtering and clustering

Feature normalisation

Normalisation and filtering has been implemented to ensure that the features in the models are comparable and robustly predictive for different irradiation settings, e.g. for different spot intensities. Absolute normalisation has been performed by subtracting the median feature value of the reference measurement (no air cavity) from each feature value:

$$x_{i,j}^{\text{abs}} = x_{i,j} - \text{median}_{\text{ref},j}, \tag{S1}$$

where $x_{i,j}$ is the feature value of the i-th spot and j-th feature and $\text{median}_{\text{ref},j}$ is the median feature value of all spots of the reference measurement (air cavity thickness = 0).

In addition, relative normalisation has been carried out by dividing this difference by the median reference value to determine the relative change:

$$x_{i,j}^{\text{rel}} = \frac{x_{i,j} - \text{median}_{\text{ref},j}}{\text{median}_{\text{ref},j}} \tag{S2}$$

Feature filtering

To ensure robustness against different irradiation settings, ten consecutive irradiated static spots were accumulated to form a test set with higher statistics. The normalised feature values were then assessed for statistical significance between the static and accumulated static spots across different air cavity thicknesses using the two-sided Wilcoxon rank-sum test. Features that showed significant differences (p-value less than 0.05) for at least one air cavity thickness were considered not robust for different spectrum statistics and excluded from the subsequent analysis.

In addition, univariate linear regression was performed to assess the importance of each feature by examining its relationship with the air cavity thickness. If the p-value of the Wald test from the linear regression was greater than 0.2, the feature was considered unimportant and was also excluded. For the energy-overarching approach (iii), feature filtering was performed independent of the individual proton energy datasets.

Feature clustering

For feature clustering, we implemented an agglomerative correlation-based clustering approach [5] to process and cluster the filtered features of a feature set. The primary objective was to reduce the dimensionality of the data while preserving its essential characteristics.

Table S1: Parameters used for the image feature extraction of the medical image radiomics processor [6].

**Parameter name**	**Value**	**Description**
discr_method	fixed_bin_size	Discretisation algorithm.
discr_bin_width	1	Bin width (in intensity units) for the fixed_bin_size algorithm.
ivh_discr_method	none	Discretisation algorithm for the intensity-volume histogram.
glcm_dist	1	Distance (in voxels) for GLCM for determining the neighbourhood.
glcm_spatial_method	2D	Calculate GLCM in 2D, 2.5D or 3D.
glcm_merge_method	average	How to treat the texture matrices.
glrlm_spatial_method	2D	Calculate GLRLM in 2D, 2.5D or 3D.
glrlm_merge_method	average	How to treat the texture matrices.
glszm_spatial_method	2D	Calculate GLSZM in 2D, 2.5D or 3D.
gldzm_spatial_method	2D	Calculate GLDZM in 2D, 2.5D or 3D.
ngtdm_spatial_method	2D	Calculate NGTDM in 2D, 2.5D or 3D.
ngldm_dist	1.8	Distance (in voxels) for NGLDM for determining the neighbourhood.
ngldm_diff_lvl	0	Difference level (alpha) for NGLDM.
ngldm_spatial_method	2D	Calculate NGLDM in 2D, 2.5D or 3D.

Table S2: Parameters used for the feature clustering method.

**Parameter name**	**Value**	**Description**
cluster_threshold	0.2	The threshold for the feature distance to determine clusters.
transformation_method	yeo_johnson	Method for feature transformation.
normalisation_method	standardisation	Method for feature normalisation.
distance_method	1 - abs(pearson)	Method for computing the correlation matrix.
clustering_method	agglomerative	Method for clustering features.
linkage_method	average	Linkage method used in agglomerative clustering.
cluster_representative_selection	highest mean absolute correlation	Criterion for selecting the most representative feature from each cluster.

Table S3: Main parameters of FAMILIAR [7] used for the feature ranking process.

**Parameter name**	**Value**	**Description**
experimental_design	bs(fs,20) + mb	Design of the experiment.
outcome_type	continuous	Type of the outcome variable.
feature_max_fraction_missing	0.01	Maximum fraction of missing values allowed in features.
filter_method	none	Method for filtering unimportant features.
transformation_method	yeo_johnson	Method for feature transformation.
normalisation_method	standardisation	Method for feature normalisation.
cluster_method	none	Method for clustering features.
fs_method	mrmr, spearman, multivariate regression or lasso	Method for feature selection.
vimp_aggregation_method	enhanced_borda	Method for aggregating variable importance.
vimp_aggregation_rank_threshold	10	Threshold for rank aggregation of variable importance.

Table S4: Main parameters of FAMILIAR [7] used for the signature determination and final model training.

Parameter name	Value	Description
model_learner	glm_gaussian, random_forest_rfsrc, xgboost_tree_gaussian or svm_eps	The model or algorithm used for learning.
experimental_design (signature determination)	fs + cv(mb,3,3)	Design of the experiment.
experimental_design (final model training)	fs + mb + ev	Design of the experiment.
outcome_type	continuous	Type of the outcome variable, e.g., continuous.
filter_method	none	Method for filtering unimportant features.
transformation_method	yeo_johnson	Feature transformation method.
normalisation_method	standardisation	Feature normalisation method.
cluster_method	none	Method for clustering features.
fs_method	none	Feature-selection methods.

Table S5: MRSE values with 95 % confidence intervals for various experiment configurations and datasets. For the single-energy subsets (i) and (ii), data from the irradiations with proton energies of 162 MeV and 225 MeV were used for model development, respectively. The energy-overarching approach (iii) combines data from both 162 MeV and 225 MeV irradiations. The specific model configuration, including feature type, feature selection method, and machine-learning model, was selected based on the best performance during cross-validation. The datasets are categorised based on the proton energy used for irradiation (162 MeV and 225 MeV) and the type of spots analysed (static vs. scanned). The specific datasets include static spots (static), single scanned spots (scanned), scanned spots accumulated over eight layers (scanned_accum8). Confidence intervals are indicated in brackets and calculated from 1000 bootstrap samples. Abbreviations: MRMR, minimum redundancy maximum relevance; MRSE, mean range shift error; XGBoost, eXtreme Gradient Boosting.

**Subsets**	**(i) 162 MeV**	**(ii) 225 MeV**	**(iii) 162 & 225 MeV**
Feature type	Combined	Combined	Time-only
Feature-selection method	Lasso	MRMR	Lasso
Machine-learning model	XGBoost	XGBoost	XGBoost
**Dataset** (spot type, proton energy [MeV])	**MRSE / mm**		
static, 162	1.89 (1.26 – 2.56)	-	1.38 (0.83 – 2.09)
static, 225	-	1.09 (0.78 – 1.36)	1.68 (1.30 – 2.14)
scanned, 162	3.84 (3.34 – 4.24)	-	3.88 (3.46 – 4.33)
scanned, 225	-	4.08 (3.63 – 4.42)	3.83 (3.36 – 4.37)
scanned_accum8, 162	1.76 (0.98 – 2.78)	-	2.12 (1.31 – 3.06)
scanned_accum8, 225	-	2.25 (1.30 – 3.29)	2.88 (2.42 – 4.22)

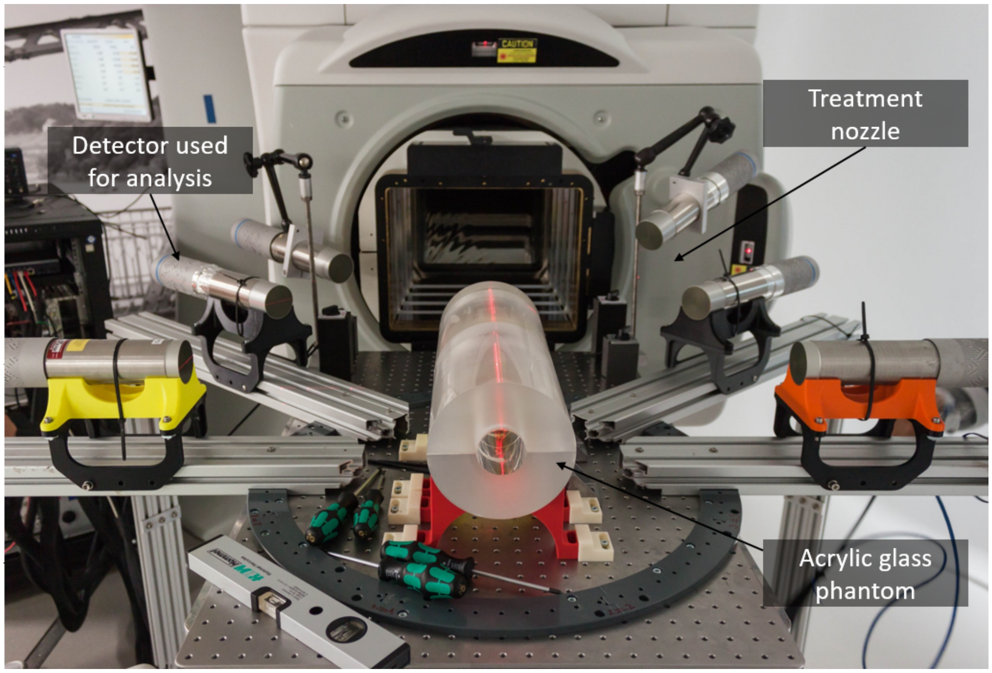

Figure S1: Photograph of the experimental setup for prompt gamma-ray detection. The setup consists of a cylindrical acrylic glass phantom (diameter: 15 cm, length: 40 cm) with adjustable air cavities ($\Delta R = 0, 5, 10, 20$ mm), and a $CeBr_3$-based detector for measuring the temporal and spectral characteristics of prompt gamma rays. The beam was delivered in static and scanned modes (on a different beam line not depicted in the image) with energies of 162 MeV and 225 MeV, and prompt gamma rays were recorded at a backward angle of 130°. The detector was positioned such that its axis intersected the beam axis at half the depth of the proton range. Further details on the experimental setup and procedures can be found in [3].

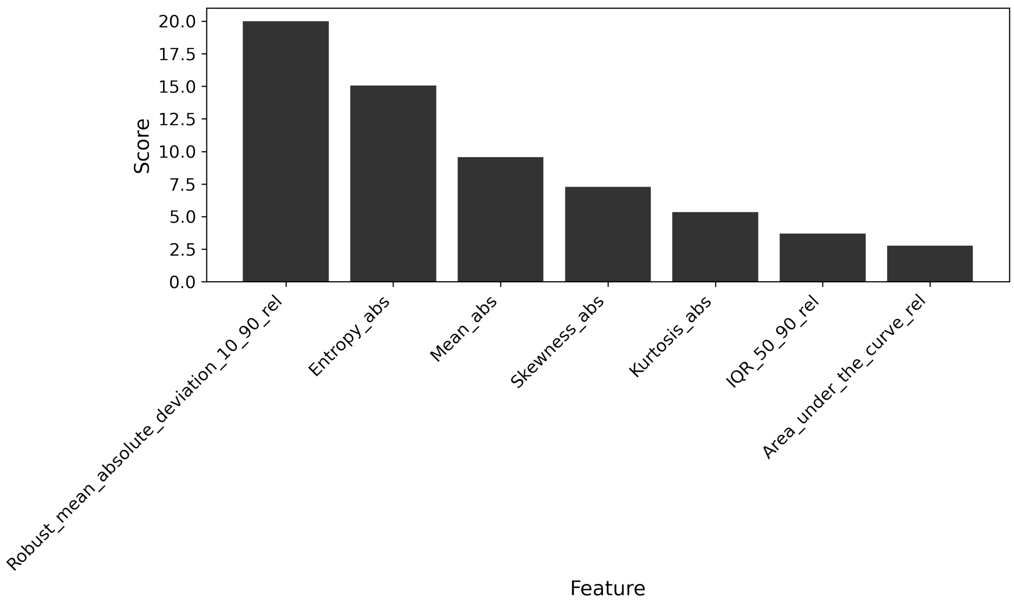

Figure S2: Bar plot showing the importance scores of time-only features (more precise cluster representatives) assigned by Lasso feature selection.

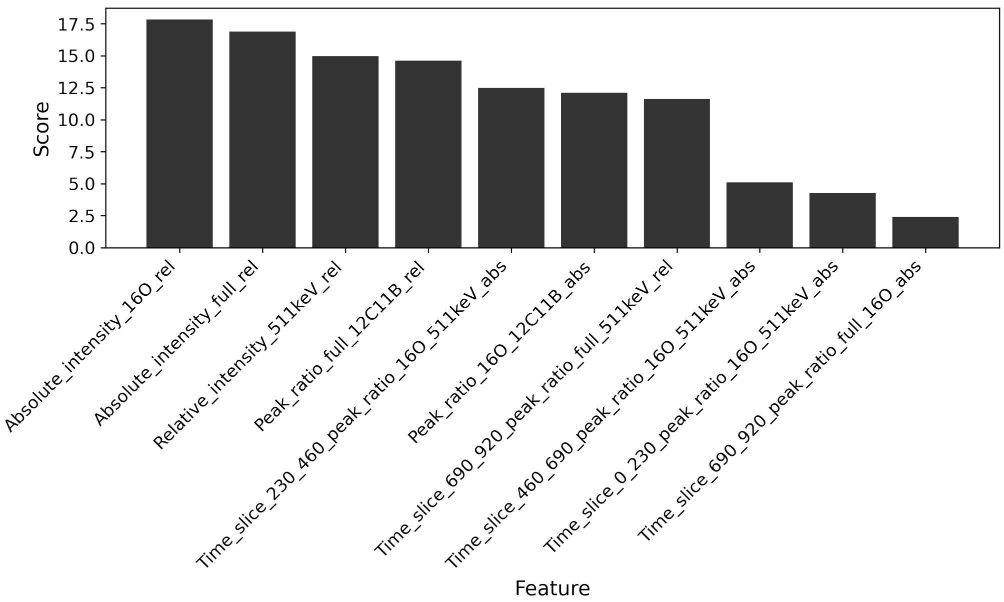

Figure S3: Bar plot showing the importance scores of energy-only features (more precise cluster representatives) assigned by Lasso feature selection.

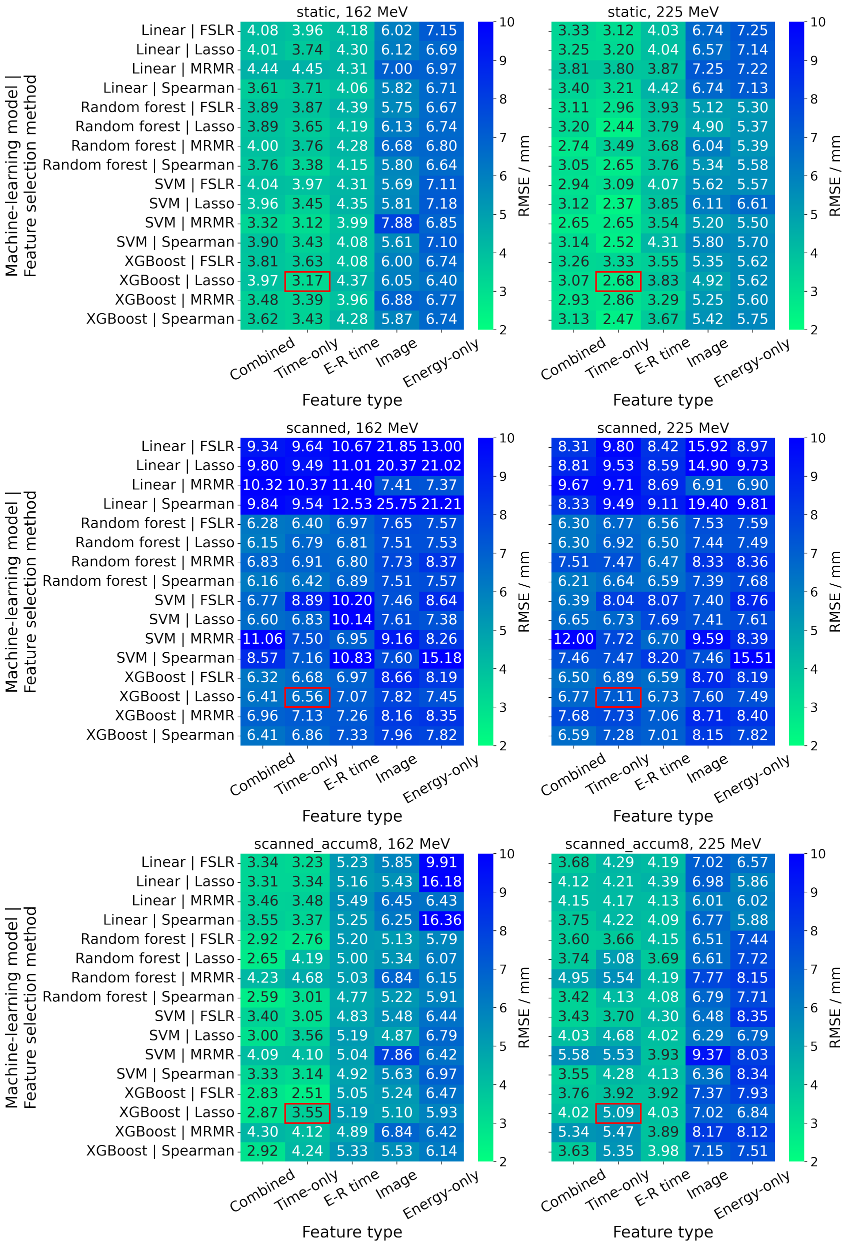

Figure S4: Test performance heatmaps for RMSE across different datasets for the energy-overarching approach. This figure displays heatmaps of the RMSE for various combinations of machine-learning models, feature types, and feature-selection methods. Each subplot represents a specific dataset. The red box highlights the performance of the final model. Abbreviations: E-R, energy-restricted; FSLR, forward selection linear regression; MRMR, minimum redundancy maximum relevance; RMSE, root mean squared error; SVM, support vector machine; XGBoost, eXtreme Gradient Boosting.

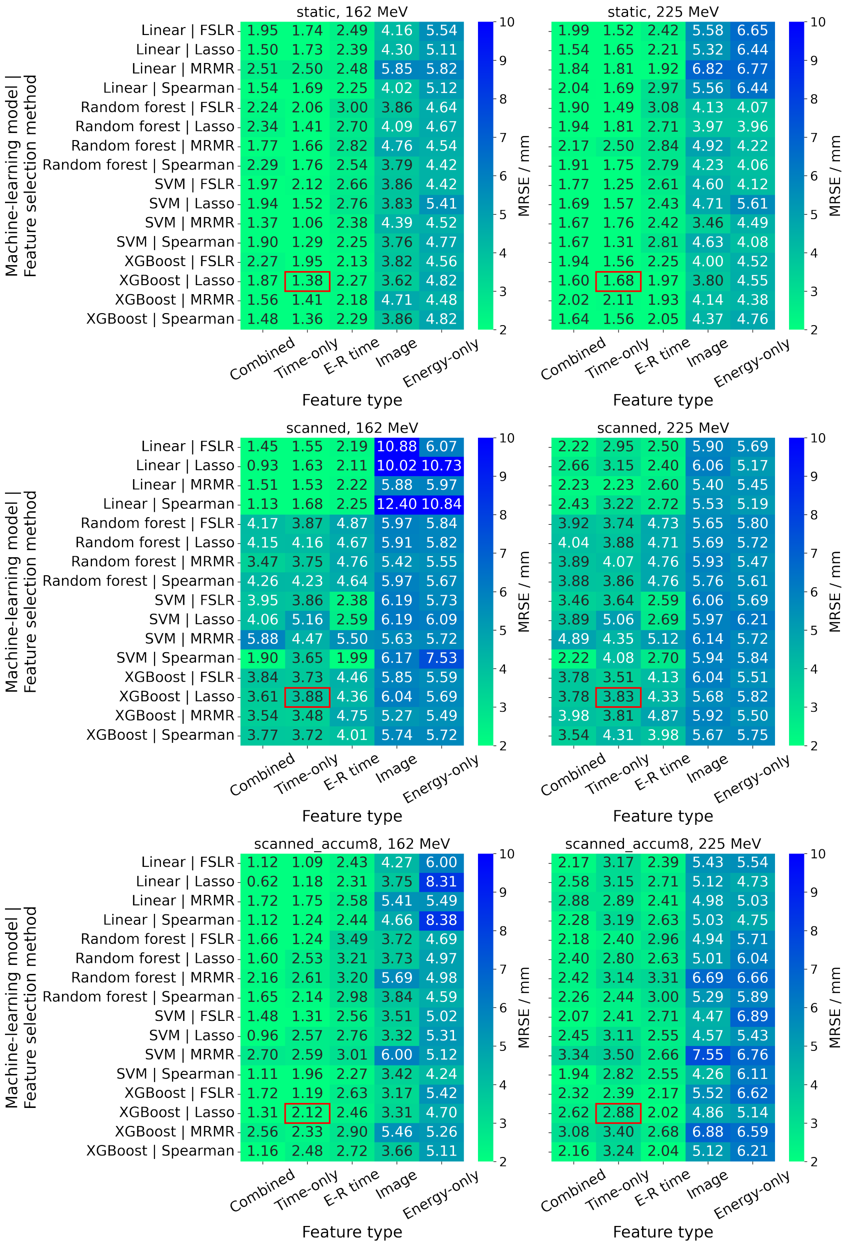

Figure S5: Test performance heatmaps for MRSE across different datasets for the energy-overarching approach. This figure displays heatmaps of the MRSE for various combinations of machine-learning models, feature types, and feature-selection methods. Each subplot represents a specific dataset. The red box highlights the performance of the final model. Abbreviations: E-R, energy-restricted; FSLR, forward selection linear regression; MRMR, minimum redundancy maximum relevance; MRSE, mean range shift error; SVM, support vector machine; XGBoost, eXtreme Gradient Boosting.

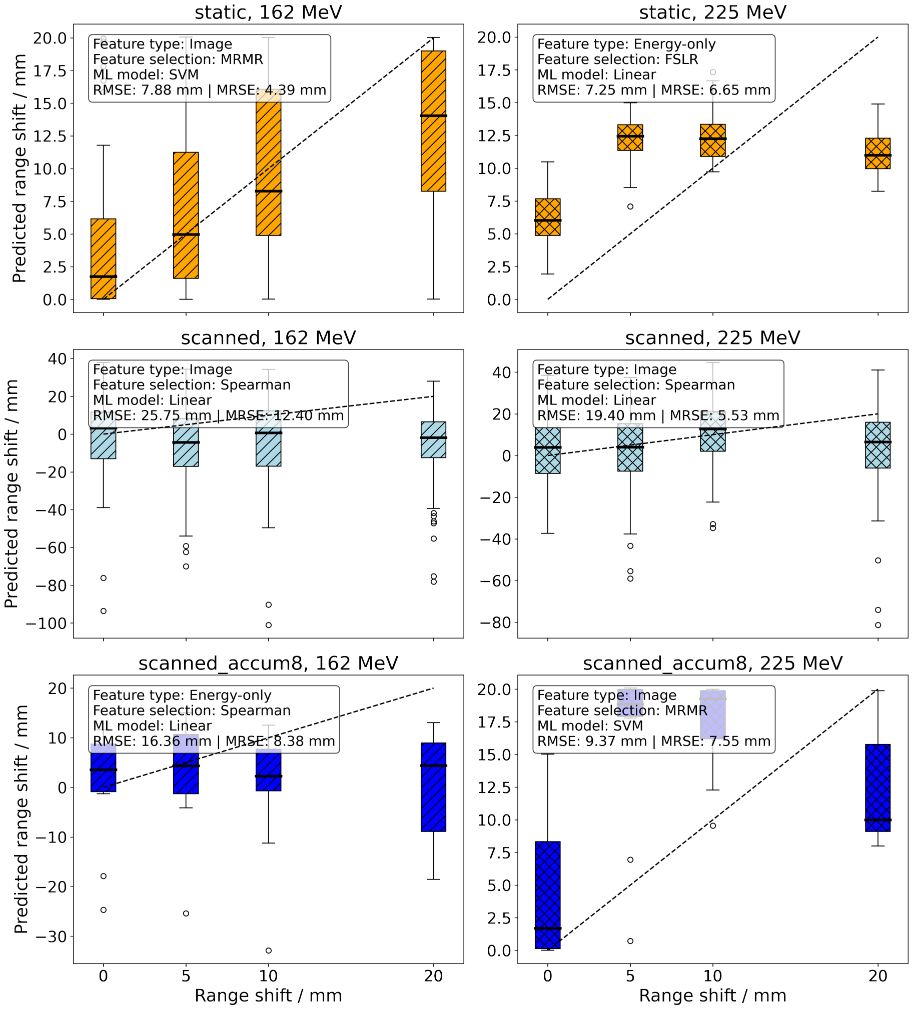

Figure S6: Boxplots of predicted range shifts for the worst-performing model configuration for the energy-overarching approach based on RMSE in each test dataset. Each subplot corresponds to a specific dataset. The black dashed line represents the identity line (ideal predictions). The box colors indicate the dataset type, with orange representing static data, light blue representing scanned data, and blue representing scanned_accum8 data. Additionally, the box textures differentiate the proton energies: "//" denotes 162 MeV and "xx" denotes 225 MeV. The model configuration, feature type, feature selection method, and RMSE/MRSE values are annotated in each subplot. Abbreviations: E-R, energy-restricted; FSLR, forward selection linear regression; ML, machine learning; MRMR, minimum redundancy maximum relevance; RMSE, root mean squared error; SVM, support vector machine; XGBoost, eXtreme Gradient Boosting.

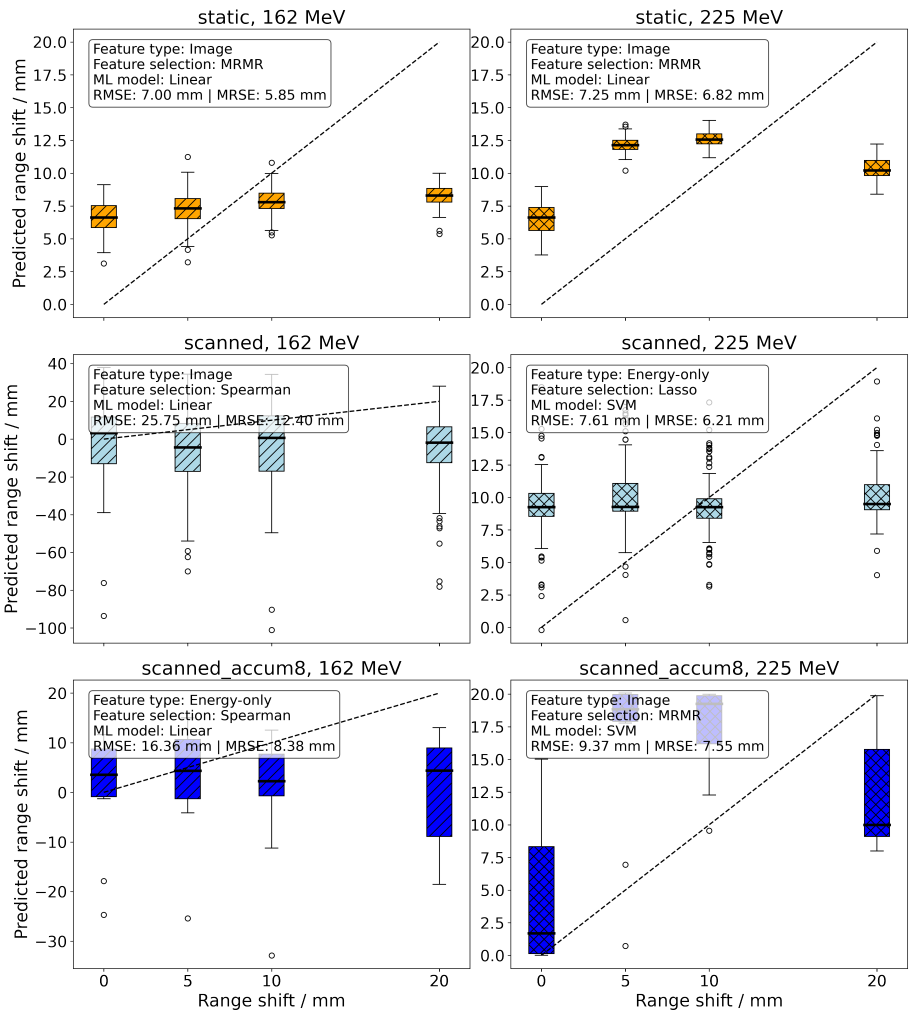

Figure S7: Boxplots of predicted range shifts for the worst-performing model configuration for the energy-overarching approach based on MRSE in each test dataset . Each subplot corresponds to a specific dataset. The black dashed line represents the identity line (ideal predictions). The box colors indicate the dataset type, with orange representing static data, light blue representing scanned data, and blue representing scanned_accum8 data. Additionally, the box textures differentiate the proton energies: "//" denotes 162 MeV and "xx" denotes 225 MeV. The model configuration, feature type, feature selection method, and RMSE/MRSE values are annotated in each subplot. Abbreviations: E-R, energy-restricted; FSLR, forward selection linear regression; ML, machine learning; MRMR, minimum redundancy maximum relevance; RMSE, root mean squared error; SVM, support vector machine; XGBoost, eXtreme Gradient Boosting.

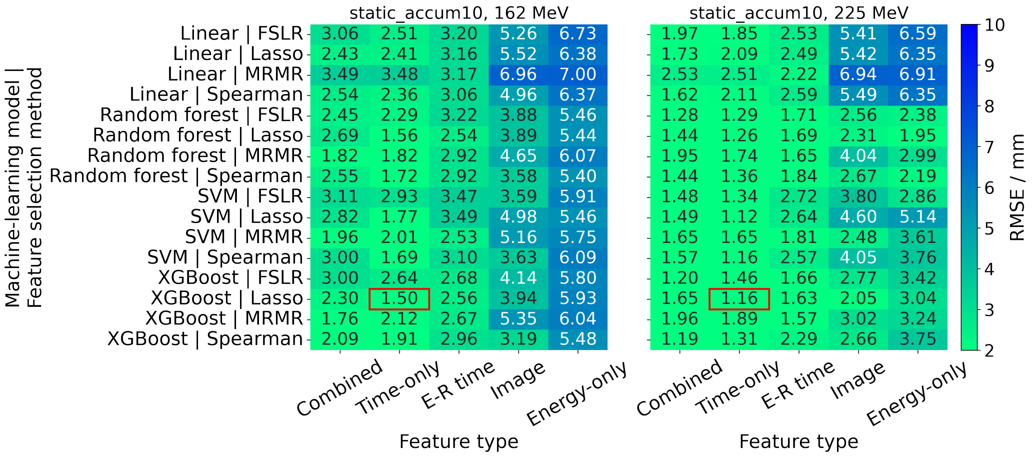

Figure S8: Performance heatmaps for RMSE across the 10-fold accumulated static spots for the energy-overarching experiment. This figure displays heatmaps of the RMSE for various combinations of machine-learning models, feature types, and feature-selection methods. Each subplot represents a specific dataset. The red box highlights the performance of the final model. Abbreviations: E-R, energy-restricted; FSLR, forward selection linear regression; MRMR, minimum redundancy maximum relevance; RMSE, root mean squared error; SVM, support vector machine; XGBoost, eXtreme Gradient Boosting.

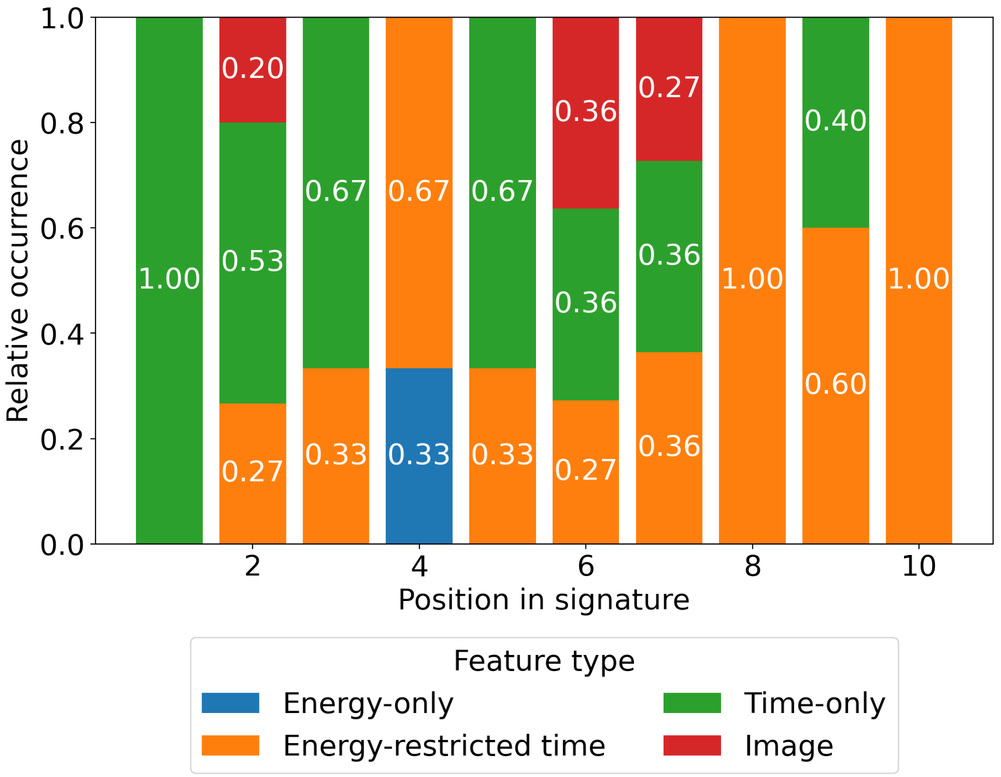

Figure S9: Distribution of feature types in the combined feature signatures for the energy-overarching approach. This figure displays the relative frequency of a feature type in the signatures of the combined feature set according to the position in the signature. Features with lower positions are considered more important. Numbers within the bars represent the respective relative frequency of the feature type.

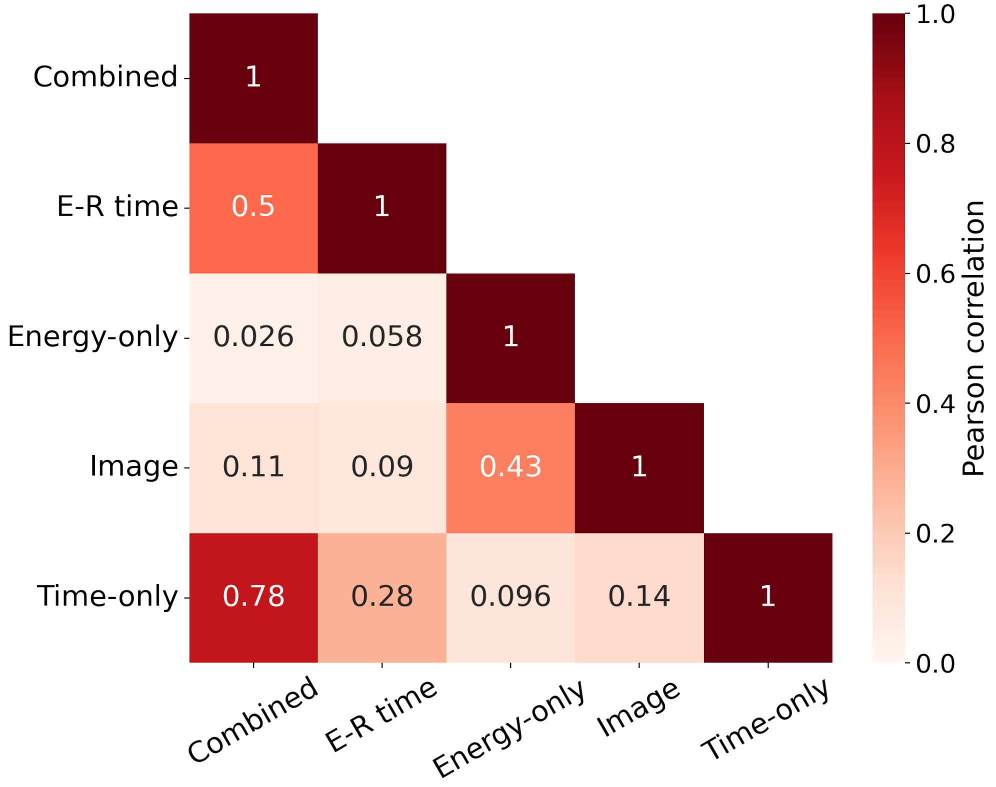

Figure S10: Average Pearson correlation of predicted air cavity thicknesses across different feature types. Each cell represents the mean correlation of predicted air cavity thicknesses between a pair of feature types, averaged across all examined air cavity thicknesses, datasets, machine-learning models, and feature selection methods. A higher correlation value (towards red) suggests stronger agreement between the predictions from models based on the respective feature types. Abbreviations: E-R, energy-restricted.

References

[1] Werner T, Berthold J, Enghardt W, Hueso-González F, Kögler T, Petzoldt J, et al. Range verification in proton therapy by prompt gamma-ray timing (PGT): Steps towards clinical implementation. In: 2017 IEEE Nuclear Science Symposium and Medical Imaging Conference (NSS/MIC); 2017. p. 1-5. https://doi.org/10.1109/nssmic.2017.8532807.

[2] Schellhammer SM, Wiedkamp J, Löck S, Kögler T. Multivariate statistical modelling to improve particle treatment verification: Implications for prompt gamma-ray timing. Front Phys. 2022;10:932950. https://doi.org/10.3389/fphy.2022.932950.

[3] Werner T, Berthold J, Hueso-González F, Kögler T, Petzoldt J, Römer K, et al. Processing of prompt gamma-ray timing data for proton range measurements at a clinical beam delivery. Phys Med Biol. 2019;64:105023. https://doi.org/10.1088/1361-6560/ab176d.

[4] Smeets J, Roellinghoff F, Prieels D, Stichelbaut F, Benilov A, Fiorini C, et al. Prompt gamma imaging with a slit camera for real-time range control in proton therapy. Phys Med Biol. 2012;57:3371. https://doi.org/10.1088/0031-9155/57/11/3371.

[5] Murtagh F. Multidimensional Clustering Algorithms. Physica-Verlag HD; 1985.

[6] Zwanenburg A, Löck S. MIRP: A Python package for standardised radiomics. J Open Source Softw. 2024;9:6413. https://doi.org/10.21105/joss.06413.

[7] Zwanenburg A, Löck S. Familiar: End-to-end automated machine learning and model evaluation. R Foundation; 2022. https://doi.org/10.32614/cran.package.familiar.
